# [Retracted] miR‑365 induces hepatocellular carcinoma cell apoptosis through targeting Bcl‑2

**DOI:** 10.3892/etm.2023.11801

**Published:** 2023-01-18

**Authors:** Mingfei Li, Yuan Yang, Yu Kuang, Xianfeng Gan, Wei Zeng, Yuping Liu, Hua Guan

Exp Ther Med 13:2279–2285, 2017; DOI: 10.3892/etm.2017.4244

Following the publication of this paper, it was drawn to the Editors’ attention by a concerned reader that the Bcl-2 protein western blotting data shown in Fig. 2D were strikingly similar to data appearing in different form in another article written by different authors who were based at different research institutes. Owing to the fact that the contentious data in the above article were already under consideration for publication prior to its submission to *Experimental and Therapeutic Medicine,* the Editor has decided that this paper should be retracted from the Journal. The authors were asked for an explanation to account for these concerns, but the Editorial Office did not receive a reply. The Editor apologizes to the readership for any inconvenience caused.

